# Clinical development of monoclonal antibody-based drugs in HIV and HCV diseases

**DOI:** 10.1186/1741-7015-11-4

**Published:** 2013-01-04

**Authors:** Michela Flego, Alessandro Ascione, Maurizio Cianfriglia, Stefano Vella

**Affiliations:** 1 Department of Therapeutic Research and Medicines Evaluation, Istituto Superiore di Sanità, Viale Regina Elena 299, 00161 Rome, Italy

**Keywords:** monoclonal antibodies, mAb-mediated antiviral mechanisms, anti-infectious biological agents, antiviral mAb based therapy, anti-HIV drugs, anti-HCV drugs, clinical studies

## Abstract

Today there are many licensed antiviral drugs, but the emergence of drug resistant strains sometimes invalidates the effects of the current therapies used in the treatment of infectious diseases. Compared to conventional antiviral drugs, monoclonal antibodies (mAbs) used as pharmacological molecules have particular physical characteristics and modes of action, and, therefore, they should be considered as a distinct therapeutic class. Despite being historically validated, antibodies may represent a novel tool for combatting infectious diseases. The current high cost of mAbs' production, storage and administration (by injection only) and the consequent obstacles to development are outweighed by mAbs' clinical advantages. These are related to a low toxicity combined with high specificity and versatility, which allows a specific antibody to mediate various biological effects, ranging from the virus neutralization mechanisms to the modulation of immune responses.

This review briefly summarizes the recent technological advances in the field of immunoglobulin research, and the current status of mAb-based drugs in clinical trials for HIV and HCV diseases. For each clinical trial the available data are reported and the emerging conceptual problems of the employed mAbs are highlighted.

This overview helps to give a clear picture of the efficacy and challenges of the mAbs in the field of these two infectious diseases which have such a global impact.

## Introduction

The innate immune response is the first-line defense in determining the outcome of an infection. Infectious agents contain conserved motifs on their surface that react with conserved pattern recognition Toll-like receptors of the host. This interaction initiates a powerful innate immune response. Moreover, the infectious agent's surface proteins and carbohydrates come into contact with B-cell receptors, membrane-bound immunoglobulin of isotype M (IgM) or D (IgD), and often induce potent antibody responses, which take some weeks to fully develop [1].

When a vertebrate organism encounters a pathogen, such as a virus or bacteria, it generates a polyclonal antibody response against numerous epitopes on different antigens during infection; therefore, polyclonal serum contains a large and diverse population of antibodies, which also include neutralizing antibodies (nAbs). Thus, polyclonal serum-derived biotherapeutic products can contain various nAbs against multiple and distinct epitopes; these nAbs provide strong protective activity due to additive or even synergistic effects on neutralization. However, in this type of product the vast majority of their constituent specific antibodies are non-neutralizing, since they are directed against misfolded protein or against epitopes on native surface proteins for which antibody binding is not protective [2,3]. Furthermore, for some viral and bacterial infections, no correlates of protection have been established; therefore, the significance of antibody titers, apart from indicating past exposure, is not clear.

Mechanisms of immunological escape can explain why total antibody titers are not always protective. Many infectious organisms, including viruses, can constantly mutate surface proteins and exploit glycans to shield important epitopes, diverting the antibody response away from functionally important epitopes in favor of immunogenic irrelevant epitopes [4].

Thanks to their protective properties, the administration of hyperimmune sera from immunized animals or immune human donors, named 'serum therapy', was the first effective treatment of infectious diseases. Later, the advent of antibiotic therapy with the advances in vaccine design has meant that serum therapy was almost abandoned for many infectious diseases. Nevertheless, hyperimmune human sera immunoglobulin preparations are still used to treat different bacterial toxins and virus related diseases, including those caused by cytomegalovirus (CMV), respiratory syncytial virus (RSV), hepatitis A virus (HAV), hepatitis B virus (HBV), rabies, vaccinia, vesicular stomatitis virus (VSV) and measles, underscoring the fact that antibody therapy remains an effective means of treatment [5,6].

**Figure 1:**
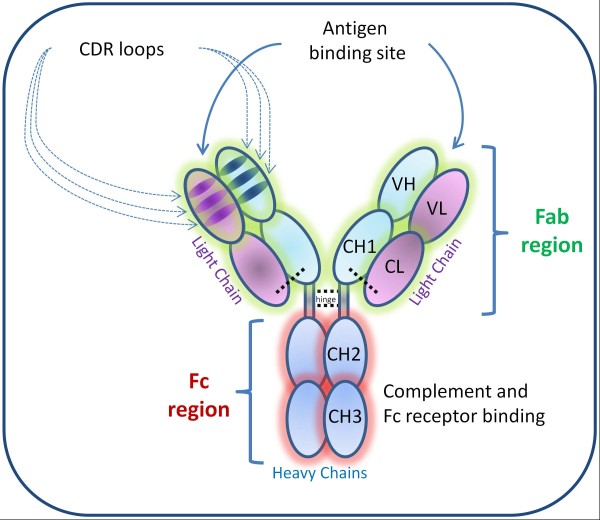
**Schematic structure of a mAb**. All immunoglobulins are composed of two identical light (L) chains and two identical heavy (H) chains, linked by disulphide bonds (black dashed bars). The heavy chains contain one variable domain (VH) and three or four constant domains (CH1, CH2, CH3 and CH4) depending on antibody isotype. By contrast, the light chains contain only one variable domain (VL) and a single constant domain (CL). Within the Fab region, at the end of the two arms of the Y-shaped molecule, the variable domain of a heavy chain pairs with the light chain variable domain to form the antigen-binding site. In more detail, within the matched V regions, three short polypeptide segments on the heavy chain and three on the light chain form the complementarity-determining regions (CDRs), which dictate the precise antigen-binding characteristics of the antibody. On the other end, the Fc domain, which includes the sites for interaction with the complement system and Fc receptors, mediates effector functions determining the fate of the bound antigen.

**Figure 2:**
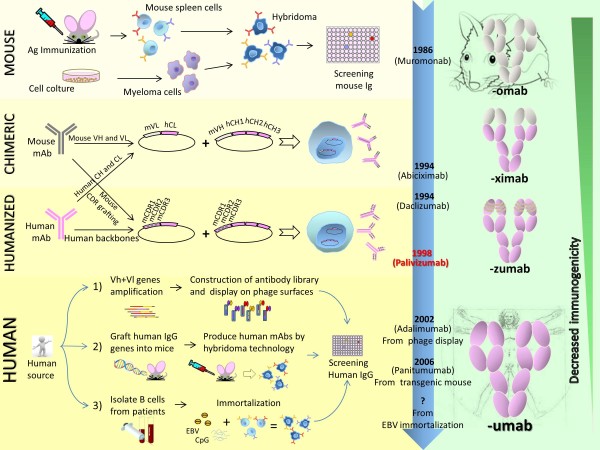
**Evolution of mAbs linked to the need to decrease their immunogenicity**. Different methods to obtain mAbs are depicted. Mouse mAbs, the 'hybridoma' cells derived from the stable fusion of immortalized mouse myeloma cells with lymphocytes from immunized mice, are screened to identify individual clones producing identical antibody to a single antigenic determinant [118]. Chimeric mAbs, the murine constant regions of both heavy and light antibody chains (mCH and mCL), are replaced with human counterparts (hCH1, hCH2, hCH3 and hCL1), leaving intact the murine variable portions (mVH and mVL) [119]. Humanized mAbs, only the CDRs of the murine mAbs (mCDRs) from both the mVH and the mVL, are 'grafted' into a human backbone antibody [120,121]. Human mAbs, 1)*Human memory B-cells *isolated from patients are immortalized by Epstein Barr Virus (EBV) and CpG oligodeoxynucleotide, and then screened for specific antibody production [122]. 2) Transgenic mice, obtained by a genetic replacement of the mouse immunoglobulin genes with human counterparts, are used to obtain fully human mAbs by traditional hybridoma technology [123]. 3) Antibody libraries, constructed by *in vitro *combinatorial assembly of human immunoglobulin variable-region gene (V genes) and cloned to provide the display onto phage surfaces, are subjected to a panning against an antigen in order to select specific clones [124]. The first mAbs of each category approved for clinical use are shown. Palivizumab is the first and, so far, only mAb approved for infectious diseases. The endings used to name the different types of mAbs are also indicated.

Today, the ability to rapidly generate and manipulate antibodies with a defined epitope recognition, named "monoclonal antibodies" (mAbs) (Figure 1), has opened a new window of opportunity for a rematch of antibodies in clinical practice. This achievement has been possible thanks to advances in cellular biology and biotechnology (Figure 2), and also to improved purification techniques which have made these therapeutics safer, less immunogenic and more effective. MAb preparations have many advantages over immune sera-derived preparations which can vary due to both time and the source of origin, since different hosts mount different antibody responses. One advantage is that mAbs, by virtue of the fact that they are chemically defined reagents, exhibit relatively low lot-to-lot variability and low risk of pathogen transmission. Another advantage for mAb preparations is the much greater activity per mass of protein since all the Ig molecules are specific for the desired target. This phenomenon is illustrated by the report that two 0.7 mg doses of two mAbs provided the same protection against tetanus toxin as 100 to 170 mg of tetanus immunoglobulins [7]. Neither does mAb therapy have the immunological complications associated with the use of heterologous sera in humans, such as serum sickness and immediate hypersensitivity, which significantly limited the latter's usefulness [8].

In recent years, mAbs have emerged as a new class of biological drugs in oncology as well as in immune and inflammatory diseases, albeit their development in infectious diseases has been slower. To date, the only mAb approved in this field is palivizumab, an anti-RSV mAb licensed for prevention of severe respiratory disease in high-risk infants and immunocompromised adults. Now the scenario is gradually changing and there are many antibodies against viruses and bacteria in various stages of clinical development. This trend has also been influenced by the development of different scientific disciplines, which makes it possible to study and dissect the function of individual microbial structures supporting the development of more targeted drugs. There are excellent reviews about this topic [6,9].

In this review we focus on the mAb-therapies now underway in clinical trials (Table 1) designed for human immunodeficiency virus (HIV) and hepatitis C virus (HCV) infectious diseases. Both these worldwide epidemics require new strategies due to the lack of a definitive cure and effective vaccines, to the continuous emergence of drug resistant variants, to the toxicities of licensed drugs and to the need to ensure a treatment for all patients. In this context, antibodies represent an intriguing alternative as therapeutics; in their favor are their different resistance mechanisms and a more favorable toxicity profile when compared to other available drug classes, fitting them for use in conjunction with the current chemotherapy by slowing the onset of resistance and possibly enhancing therapeutic efficacy.

**Table 1:** Anti-HIV and HCV mAbs in clinical development.

Compound	Target	Origin	Company	Indication	
**MAbs against viral antigens**					

3 mAb cocktail C2F5, C2G12, and C4E10	Anti-Gp41, anti-glycan structure of Gp120	Human mAb	Rockefeller University	Therapy of HIV infection Phase I/II	
P2G12	Anti-glycan structure of Gp120	Human mAb	University of Surrey European Commission	Prevention of HIV infection Phase I	
KD-247	Anti-V3 loop of HIV-1 Env	Humanized mAb	The Chemo-Sero-Therapeutic Research Institute	Therapy of HIV infection Phase I	
F105	Anti-Gp120, CD4 binding site	Human mAb	National Institute of Allergy and Infectious Diseases (NIAID)	Therapy of HIV infection Phase I	
MBL-HCV1	Anti-E2	Human mAb	MassBiologics	Prevention of liver reinfection with HCV after transplantation Phase II	

**MAbs against host antigens**					

IBALIZUMAB	Anti-CD4 receptor	Humanized mAb	TaiMed Biologics/Tanox	Therapy of HIV infection Phase I/II	
PRO-140	Anti-CCR5 receptor	Humanized mAb	Progenics/PDL	Therapy of HIV infection Phase I/II	
CCR5mAb004	Anti-CCR5 receptor	Human mAb	Human Genome Sciences	Therapy of HIV infection Phase I	
BAVITUXIMAB	Anti-phosphatidyl serine.	Chimeric mAb	Peregrine Pharmaceuticals	Therapy of HIV/HCV co-infection Phase II	Therapy of chronic HCV infection co-administrated with ribavirin Phase II

**Immunomodulatory mAbs**					

TREMELIMUMAB CP 675,206	Anti-CTLA-4	Humanized mAb	Pfizer	Therapy of HIV infection Phase I (study withdrawn prior to enrollment)	Therapy of advanced HCC in HCV infected patient Phase II
CT-011	Anti-PD-1	Humanized mAb	CureTech	Therapy of chronic HCV infection Phase I/II	
BMS 936558 (MDX1106)	Anti-PD-1	Human mAb	Bristol-Myers Squibb/Medarex	Therapy of chronic HCV infection Phase I	

### MAbs-mediated clearance of viruses and infected cells

The antibody structure comprises a pair of identical heavy and light chains linked by disulphide bonds held in a Y-shaped arrangement (Figure 1). The fragment antigen-binding (Fab) portion, the region that binds the antigen, is composed of one variable and one constant domain of both the heavy and the light chain. The remaining constant sections of the longer heavy chains form the tail of the Y, termed the crystallizable fragment (Fc) region, which provides the signal for effector functions.

Antibodies can provide protective effects through various mechanisms [10]. Viral neutralization is generally meant as the ability of an antibody to provide sufficient steric interference to disrupt the interaction between a microbic antigen and its ligand in experimental conditions *in vitro*. This activity is clearly associated with protection, thanks to their Fab domain alone, both in natural infection and after immunization. Virus infection includes sequential steps beginning with attachment to cell-surface receptors and ending with delivery of the viral genetic material into the cytoplasm [11]. Fusion of viral and cellular membranes is a basic entry mode for enveloped viruses, such as HIV and HCV [12], which still differ in specific aspects of viral entry and assembly, thus offering unique therapeutic opportunities. The cell surface is certainly more directly accessible for the action of the antibodies; therefore, the phase of virus entry is one of the most important targets in preventing viral infection at the origin, and many known nAbs act at this step. For the same reason, inhibition of the release of progeny virus is another possible mechanism of neutralization, as demonstrated by antibodies directed against influenza A virion surface neuraminidase [13]. In HIV and HCV fields, no virus release inhibiting antibodies have been identified to date.

**Figure 3:**
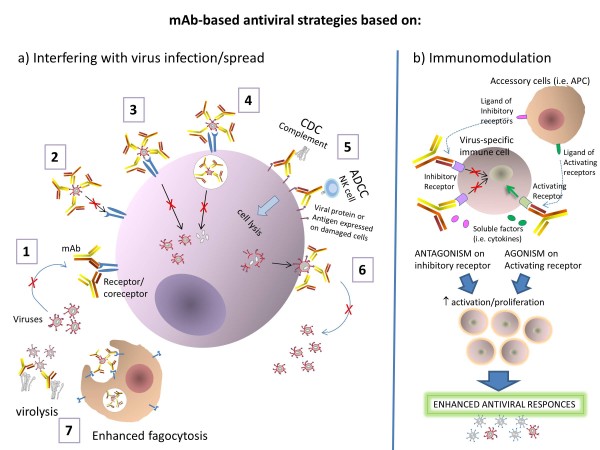
**Possible mechanisms of action of the mAbs with antiviral properties**. Panel **A **shows a hypothetical viral life cycle, highlighting potential points for therapeutic antiviral mAbs. Antibodies can block receptor engagement by binding to specific virus surface proteins (1), as well as by binding to the viral receptor or co-receptor on host cell surface (2). Some antibodies, can neutralize viral infection through interfering with conformational changes required for membrane fusion and subsequent release of the viral core into the target-cell cytoplasm; this post-binding neutralization may occur at the cell surface (3), or inside the endosomes for the viruses (for example, HCV) whose entry into the cell requires an endocytosis step (4). Antibodies recognizing viral or host proteins expressed on infected cell surface can exert protective actions through the Fc-mediated effector functions (for example, CDC, ADCC) (5). Again, mAbs may prevent the release of progeny virions (6). At the bottom the antibody neutralizing effects on the viruses before cell binding, including the direct virolysis by CDC and the mAb-mediated enhanced phagocitosis, are shown (7). In Panel **B**, the possible mAb-mediated immunomodulary therapies are depicted. In some chronic viral infections, virus-specific immune cells may persist in a 'non-functional' state, because of an imbalance of immunoregulatory signals involving multiple inhibitory and activating receptors, triggered by soluble factors and/or cell surface ligands. Therapeutic approaches using specific mAbs to block host immunosuppressive molecules (antagonism) or to trigger activating receptors (agonism) may be a valid strategy to restore immune cell function and treat various chronic viral infections.

The interaction of HIV envelope surface protein gp120 with its host receptor, CD4, on human T cells triggers conformational changes in the envelope, resulting in exposure of a transient binding site for co-receptor CCR5 or CXCR4. This in turn promotes additional conformational changes in virus gp41 protein which allow it to insert its fusion peptide into the target cell membrane to initiate membrane fusion and viral entry into host cells. NAbs can inhibit viral infection by several different mechanisms in parallel with the steps that allow the viruses to enter into cells (Figure 3). They can directly block virus attachment to target cells by interfering with virus-receptor interactions, as in the case of nAbs against the CD4-binding site on HIV gp120 [14]. This same goal can also be achieved by directing the antibodies to the virus receptor and/or co-receptor on host cells. MAbs can also block fusion at the cell membrane at the post-binding/pre-fusion stage, as exemplified by anti-CD4 [15] and/or anti-CCR5/CXCR4 (CC-motif receptor 5/CXC-motif receptor 4) mAbs, under development [16]. Again, mAbs directed to the external proximal membrane region of HIV gp41 can interfere with conformational changes needed for membrane fusion [17].

Unlike HIV, HCV entry into target cells occurs via clathrin-mediated endocytosis of the viral particle [18]. Subsequent release of the viral genome into the cytosol requires the pH-dependent fusion of viral and cellular membranes. Current models suggest that HCV circulate as LipoViral-Particles (LVPs) in the vascular system, these consisting of lipoproteins in complex with virus particles. Following localization to the surface of hepatocytes through interactions of LVPs with glycosaminoglycans and the low density lipoprotein receptor, specific binding of the E1 and E2 virus surface glycoproteins with the host SR-B1 scavenger receptor and CD81 occur [19]. Subsequently, viral particles are translocated to regions of the membrane possessing tight junction proteins occludin and claudins; the binding to these receptors results in clathrin-mediated endocytosis. As for HIV, mAbs directed against spike viral proteins, as well as against host receptors, may act at an early stage of infection by preventing the binding of the virus on the cell surface. For example, antibodies recognizing the CD81-binding site within the envelope glycoprotein E2 have been shown to block viral entry, as have a number of anti-receptor antibodies targeting CD81 [20,21] and SR-BI [22]. Some antibodies may act by blocking conformational changes and/or the requisite interactions between the viral and endosomal membranes required for fusion; although as yet no fusion determinant within the envelope glycoproteins has been defined.

Host protection *in vivo *is more complex and involves the interaction of antibodies with cells and molecules of the innate immune system. The antibody can exert protective actions through an Fc region-mediated recruitment of other components of the immune system, including antibody-dependent cell-mediated cytotoxicity (ADCC), complement-dependent cytotoxicity (CDC) and antibody-dependent cellular phagocytosis. Receptors for the Fc segment of IgG (Fcy receptors; FcγRs) are expressed on the surface of different types of cells, including natural killer cells (NK), monocytes, macrophages, dendritic cells and neutrophils. With the exception of γδT cells, FcγRs are not normally found on T lymphocytes. Similarly, the receptor for Fc segment of IgA, the FcαR, involved in phagocytosis and induction of microbe killing, is expressed on monocytes, macrophages and neutrophils [23].

The ADCC process is triggered by the interaction between the Fc region of an antibody bound to a non-self antigen exposed on host cells, and the Fc receptors on immune effector cells. The subsequent release of cytokines and cytotoxic granules containing perforins and granzymes promotes the death of the target cell. CDC is initiated by complement component C1q binding to the Fc region of IgG, which is in turn bound to the foreign antigen on the cell surface. This triggers a proteolytic cascade to activate the complement, so leading to the formation of a membrane attack complex that kills the target cell by disrupting its cell membrane. The Fc region can also mediate complement binding to and deposition on free virions, which can cause a direct virotoxic effect or inhibit virus binding to cells. Moreover, the so-called opsonization process, consisting of the binding of antibody Fab portion to the antigen following by the interaction of Fc domain to an Fc receptor on phagocytes, is a powerful mechanism to enhance the phagocytosis [9].

With respect to HIV, a potential role for ADCC in modulating the course of HIV infection was first proposed on the basis of studies showing an inverse association between ADCC antibody levels and the clinical stage of disease. The strongest evidence for a role for ADCC antibody in disease progression comes from a study by Baum *et al. *of the Multicenter AIDS Cohort Study [24]. In that study, rapid progressors had significantly lower ADCC antibody titers against CEM. NKR cells coated with gp120 than did non-rapid progressors at corresponding visits or non-progressors at any visit. Morever, HIV-infected individuals with spontaneously undetectable viremia were shown to have higher ADCC antibody levels than viremic subjects [25].

In the context of HCV infection, Fc-mediated effector functions, although less well understood, can still have an important role. Sera from both the acute and chronic phase of infection can mediate ADCC via binding to viral protein E2 expressed at the cell surface [26], while several E2-specific mAbs are able to induce CDC of E2-expressing cells [27]. Optimizing non-nAb effector functions, such as ADCC, CDC and fagogocytosis, may prove critical in the design of new effective anti-HCV therapeutic antibodies [28].

### Modulation of immune response by mAbs in order to overcome exhaustion in chronic viral infections

Many viruses, including HIV and HCV, have developed mechanisms for evasion and/or modification of the host's innate and adaptive immune response, often causing persistent viral infection. One of the most extensively investigated examples of evasion of the host's adaptive immune system is the exhaustion of virus-specific T cells. Exhaustion consists of a progressive dysfunction characterized by the inability to proliferate and to produce key antiviral and immune stimulating cytokines (for example, interleukin (IL)-2, tumor necrosis factor (TNF)-a, interferon (IFN)-γ), or to lyse infected cells [29].

A feature of functional exhaustion is that it affects many antiviral properties of both mouse and human CD8+ T cells. Loss of effector functions proceeds in a hierarchical manner starting with defects in IL-2 production and proliferation, followed by the decrease of TNF production. Cytotoxic activity is also lacking in exhausted human CD8+ T cells. At a severe stage of exhaustion, IFN-γ production is eventually compromised, with exhausted T cells ending up deleted if the high antigenic load persists [30]. Exhaustion can also occur in CD4+ T cells in both mice [31] and humans [32]. Probably the best explanation for this progressive dysfunction and loss of effector T cells is the continuous triggering of virus-specific T cell receptors owing to a high antigenic load in persistently infected hosts without a critical rest period. The current consensus is that functional exhaustion is a way of limiting the magnitude of effector T cell responses. Although this may safeguard against autoimmune responses, it may also compromise effective immunity against persistent infectious agents and tumors [29].

Exhausted T cells are subject to complex layers of negative regulation. This involves signaling through multiple inhibitory receptors that inhibit functional and proliferative responses. The CD28 family member programmed cell death 1 (PD-1) has been shown to be the most highly expressed inhibitory receptor on CD8+ T cells during chronic infection, and to have a major role in regulating T cell exhaustion during infection [33,34]. Increased expression of PD-1 by T cells also occurs during HBV and HCV infections [35-37]. Several other inhibitory receptors have also been shown to induce T cell unresponsiveness during chronic infections. These receptors include cytotoxic T lymphocyte antigen 4 (CTLA-4) [31,38,39], T cell immunoglobulin domain and mucin domain protein 3 (TIM3) [40,41], and lymphocyte activation gene 3 (LAG-3) [38]. In addition, certain cytokines, such as IL-10 and transforming growth factor-β (TGFβ) as well as regulatory T cells, may also contribute to the lack of T cell functionality during situations of high antigenic burden [42].

There is intriguing evidence that blockade of the inhibitory receptor could restore antigen T cell responses. For example, blockade of the PD-1 signaling pathway improves antigen-specific T cell proliferation and cytokine secretion in lymphocytic choriomeningitis virus (LCMV)-infected mice [31,34] and in humans with chronic HIV [32,43,44], HBV [45] and HCV [36] infections. This effect was synergistically improved in LCMV infected mouse following the simultaneous blockade of the T cell inhibitory receptors PD-1 and LAG-3, thanks to which a diminished viral load *in vivo *was observed, although blocking LAG-3 pathway alone had little effect on the severity of exhaustion [34]. Moreover, mAb-mediated blocking of CTLA-4 pathway *in vitro *augments HIV-specific CD4+ T-cell function suggesting that the immune modulation of this target may also provide a clinical benefit in infected individuals [39]. Another example is the manipulation of signals mediated by glucocorticoid-induced TNF receptor (GITR), a recently identified member of the TNF receptor superfamily, preferentially expressed on subset CD4+CD25+ regulatory T cells. GITR signals break the suppressive activity of this subset. In fact, an agonistic anti-GITR mAb immediately injected after viral infection significantly increased the number of activated CD4+ and CD8+ T cells secreting IFN-γ [46].

One must remember that the manipulation of immunological responses could have detrimental effects on the host, as highlighted by the recent tragic human trial of TGN1412. This is a mAb against human T cell co-stimulatory molecule CD28 developed by TeGenero to treat B-cell chronic lymphocytic leukaemia, autoimmune and inflammatory diseases, on the basis of its capability of inducing preferential activation and expansion of immunosuppressive regulatory T (Treg) cells, as observed in rodent models. TGN1412 has been termed a 'superagonist' because it binds to CD28 and activates T cells without the need for prior T cell antigen receptor (TCR) signaling. In a Phase I clinical trial (in March 2006), following administration of TGN1412, six healthy young men suffered a life-threatening cytokine-release syndrome (CRS) involving multi-organ failure, something unpredicted by the preclinical studies. It is now clear that in the presence of TGN1412, activated CD4+ effector memory T (T_EM_) cells were the source of the cytokines that mediated the CRS observed in the volunteers. Treg cells were not able to prevent systemic inflammation, probably because the balance between activated Treg cell and T_EM _cell numbers is disadvantageous for humans compared with laboratory rodents. Furthermore, in macaques, but not in humans, CD4+ T cells lose CD28 expression during their differentiation into T_EM _cells; this detail, however, had gone unnoticed despite many years of primate testing. In conclusion, this model failed to prevent the disastrous case above [47].

In view of these events, such a risk needs to be carefully assessed if the modulation of immune inhibitory or activating receptors is used for increasing the functional activity of virus-specific T cells in order to avoid non-specific inflammation. These therapeutic approaches are being carefully evaluated for cancer as well as for HIV and HCV chronic viral disease.

### Clinical development of anti-HIV and HCV mAbs

#### MAbs against viral antigens

Given the potential antiviral effect of the antibodies, viruses have evolved multiple mechanisms to protect themselves from antibody binding. One of these, the viral receptor glycosylation, is widely shared among different viruses. Carbohydrates are poorly immunogenic and, therefore, do not stimulate the response of type B lymphocytes and simultaneously hide the underlying protein structures. HCV E2 protein contains up to 11 potential N-linked glycosylation sites. Specific glycans mask the CD81-binding site and, therefore, nAb epitopes [48]. Lipid shielding may represent an additional strategy used by HCV to evade the antibody response. Current data suggest that key neutralizing epitopes are less accessible on LVPs. More recently, HCV has been found capable of direct cell-to-cell transmission, which is largely resistant to antibody neutralization [49,50]. HIV envelope protein is also glycosilated and changes occur in the frequency and position of glycans HIV gp120; these 'evolving glycan shields' have been shown to decrease sensitivity to antibody neutralization [51]. Other factors of antibody escape for HIV are: trimerization of the gp 120 and gp 41 that can shield vulnerable epitopes better exposed on the individual monomeric subunits; kinetic and spatial constraints that impede antibodies from accessing potentially vulnerable sites during receptor binding and membrane fusion process; the variable loops of gp120 that are a prime target for nAbs, which usually have a very narrow breadth of reactivity [52]. Finally, the high mutation rate of many viruses, including HIV and HCV, which undergo rapid antigenic variation, allows them to escape neutralization, constituting a significant hurdle for nAbs development.

All these problems may be counter-balanced by selecting nAbs which target conserved and more accessible areas of viral particles, and/or by using mixtures of nAbs which target various key epitopes. In fact, it has been demonstrated that combination therapy with mAb cocktails prevents escape variants for many viruses, including influenza [53], coronavirus [54] and LCMV [55], and that broad neutralization in the sera of most of some individual HIV infected donors can be associated with single or four to five principal specificities [56].

Recent studies have indicated that nAbs play a critical role in HCV disease outcome. Viral clearance was associated with a rapid induction of neutralizing antibodies in the early phase of infection with some evidence that these antibodies are broadly reactive [57,58]. In contrast, chronic HCV infection was characterized by absent or low-titer neutralizing antibodies in the early phase of infection and the persistence of infection, despite the induction of cross-neutralizing antibodies in the later phase of infection. Current understanding of the nAb response raised against HCV suggests that E2 is the major target, and that multiple epitopes within E2 may be targeted by both linear-and conformation-dependent antibodies. Predominantly, these neutralization epitopes overlap with CD81-binding sites and clearly demonstrate a role in inhibition of entry. Currently, one of these mAbs, MBL-HCV1, is being investigated in clinical trials in the prevention of liver re-infection after transplantation, for which novel antiviral preventive and therapeutic strategies are urgently needed. In fact, re-infection of the graft is universal, being characterized by accelerated progression of liver disease; IFN-based therapies exhibit enhanced adverse effects and limited efficacy in these patients [59,60]. MBL-HCV1 is a fully human monoclonal antibody isolated from transgenic mice and directed to a highly conserved linear epitope of HCV E2 glycoprotein. It is able to neutralize pseudoviruses from multiple HCV genotypes and has demonstrated efficacy in preventing HCV genotype-1 infection in HCV naïve chimpanzees. A phase I open-labeled, dose escalation study was performed in healthy adult volunteers starting with 1 mg/kg and escalating to 3, 10, 30 and 50 mg/kg after a 10-day post-infusion safety review. MBL-HCV1 was well-tolerated without any seriously adverse effect event. Based on the favorable safety, tolerability and pharmacokinetics data, a phase II study of MBL-HCV1 in chronically infected HCV patients undergoing liver transplantation has been planned [61].

In the context of HIV disease, despite intensive study over two decades, only a small number of broadly neutralizing mAbs have been identified from infected patients and little is known about their activity *in vivo*. These antibodies are able to inhibit viral entry of most primary HIV isolates *in vitro *[17,62-64] and the exceptionally high level of mutation found in their genes may reflect chronic immune responses to HIV and persistent hypermutation and selection [65]. A number of trials evaluating different formulations of anti-HIV monoclonal antibodies are now in progress.

The first trial assessed a chimeric monoclonal antibody CGP 47,439 to the V3 loop of the HIV-1 envelope gp120 over 21 weeks [66,67]. Subsequent studies evaluated the kinetics of monoclonal antibody F105 directed to the CD4-binding site of gp120 [68,69], a humanized antibody binding to the V3 epitope GPGRAF [70]. Finally, a humanized mAb, KD-247 is under evaluation in clinical trials. Its epitope was mapped to 6 aa, IGPGRA, at the tip of the V3 loop of Envelope protein and demonstrates cross-neutralizing activity against HIV-1 isolates in clade B [71].

A drug based on the mAb cocktail mode is also currently in clinical development. In this regard it has already been observed that in HIV neutralization assays the effectiveness of a mix of broadly neutralizing antibodies increased synergistically compared to the effect of the individual antibody. The synergy effect was relatively weak, with a maximum of two- to four-fold enhancement, between antibody pairs, thereby increasing neutralization titers about 10-fold in triple and quadruple antibody combinations [72]. However, the use of antibodies in the cocktail mode, as an approach to improve their effectiveness, is already recognized for other pathogens or toxins. In the case of tetanus toxin, it has been reported that combining the action of three out of four antibodies increased the neutralizing activity up to 200 times [73]. In the case of botulinum toxin, neutralizing activity has been reported up to 20,000 times higher when using a mixture of three monoclonal antibodies [74]. Instead, other studies have demonstrated that the combination of two potent neutralizing mAbs against HIV, VRC01 and PG9, although not synergistic, can mediate additive neutralization viral activity and provides an improved neutralization coverage of 90% to 97% of viral strains by combining independent epitope targeting [75].

In a proof-of-concept passive immunization trial with humans, it has been demonstrated that a cocktail of the three broadly neutralizing mAbs - 2G12, 4E10 and 2F5 - was able to delay viral rebound in patients whose infections were fully suppressed by antiretroviral treatment before administration of the antibodies [76]. Interestingly, the main antiviral effect observed was primarily attributable to the 2G12 antibody, a mAb that binds to a non-continuous epitope composed of glycosylation residues distributed over the envelope protein gp120 [64], whereas the other two mAbs, 4E10 [77] and 2F5 [78], recognize two adjacent highly conserved epitopes on the membrane-proximal ectodomain of the HIV-1 envelope protein gp41. In earlier phase I clinical trials, safety and tolerability were demonstrated [79,80]. During a long-term multiple dose phase II clinical trial, high doses of the three neutralizing antibodies were given in combination to 14 HIV-1-infected individuals at weekly intervals over three months. Pharmacokinetic analysis revealed that repeated infusions at high dose levels were well tolerated by the patients and did not elicit an endogenous immune response against the monoclonal antibodies. The antibodies showed distribution and elimination kinetics similar to those seen for other human-like antibodies, though monoclonal antibody 2G12 had a significantly longer elimination half-life (21.8 +/- 7.2 days) than monoclonal antibodies 4E10 (5.5 +/- 2.2 days) and 2F5 (4.3 +/- 1.1 days) [81]. Furthermore, analyses of the emergence of mutations conferring resistance to these three mAbs were performed. Sequence analysis of the 2G12 epitope relevant N-glycosylation sites of viruses derived from 13 patients demonstrated that mutations in these sites are associated with resistance. *In vitro *selection experiments with isolates of four of these individuals corroborated the *in vivo *finding that virus strains rapidly escape 2G12 pressure. Importantly, *in vitro *selection with 2F5 and 4E10 demonstrated that resistance to these nAbs can be difficult to achieve and can lead to selection of variants with impaired infectivity [82]. Moreover, generation of viruses resistant to the triple-combination was a slower process characterized by recurrent loss of virus replication; some generated triple-resistant viruses seemed to be impaired in their replicative fitness, and none of the patients developed detectable viruses that escaped neutralization by all three mAbs within the 77-day observation period [83].

As is true with all mAbs designed for infectious disease, the development of a successful vaccine would reduce the need for them. However, given the scarcity of drugs in the field of virology and given the slow progress on the HIV vaccine front, the development and use of microbicides, compounds that could be applied topically to prevent HIV transmission, is one of the possible strategies to counter the spread of HIV. In this regard, mAbs could be proposed as suitable components of microbicides to fight HIV entry at mucosal surface. A safety study of P2G12 mAb administered vaginally in healthy women has been completed. P2G12 is the broadly neutralizing 2G12 mAb manufactured from tobacco plants [84]. Most mAbs in clinical trials have been produced using a system called Chinese Hamster Ovary cell (CHO-Cell) fermentation [85], including 2G12 used along with 2F5 and 4E10 antibodies as a cocktail. The CHO-Cell fermentation production method is very expensive and cannot produce enough mAbs on a scale required for the global market; therefore, plant manufacture of such mAbs may hopefully offer some solutions to lower production costs and improve output. The process yields five grams of purified antibody from 250 kg of tobacco and production costs could be 10 to 100 times lower than when using conventional bioreactors. This study has been designed to confirm the safety of a vaginally delivered mAb P2G12 derived from plants and manufactured to Good Manufacturing Practice (a quality standard used for the manufacture of medicinal products). The medicine is the first plant-produced antibody to be greenlit for clinical testing by Britain's Medicines and Healthcare products Agency (MHRA). It took about a year to get that agency's stamp of approval because it required assurances that the drugs did not contain allergenic plant sugars or pesticides. No matter how it is produced, P2G12 antibody has not been shown to actually prevent HIV-1 infection in clinical trials; thus a version made from tobacco plants would not see approval any time soon. P2G12 would also likely be just one ingredient in a cocktail of plant-produced antibodies [84].

#### MAbs against host receptors

To eliminate or reduce the development of escape variants it has been proposed that targeting the conserved cellular receptors of the virus may open new avenues for a viable antibody therapy for HIV infection. HIV entry into CD4+ T cells requires the presence of a co-receptor, either CCR5 or CXCR4, on the target cell. Thus, based on this hypothesis, mAbs directed against CD4 and against the co-receptor CCR5, have been developed and are being analyzed in clinical trials.

CD4 functions as a co-receptor, physically associating with the TCR during Ag recognition by binding to a non-polymorphic component of the major histocompatibility complex (MHC) class II molecules on the surface of the antigen-presenting cell. Ibalizumab, a humanized mAb, binds CD4 on T cell surface away from the binding site for MHC class II molecules. It does not inhibit gp120 binding to CD4 but appears to exert its antiviral property by post-binding conformational effects that prevent CD4-bound gp120 from interacting with CCR5 or CXCR4 [19,86]. By contrast, other monoclonal antibodies, that competitively inhibit gp120 binding, interfere with MHC class II immune function [87,88]. The reported human experience with Ibalizumab consists of three clinical trials. During phase I study, it was observed that peak mean reductions in viral load occurred later in the higher dose cohorts, whereas the extent and duration of viral suppression correlated with the degree of CD4+ cell coating by ibalizumab, which was maintained longer in the higher dose cohorts, with a duration of 15 to 34 days. Peak increases in CD4 counts at one day after infusion, well before the peak declines in viral load; this suggests that the increase may have been due to redistribution of CD4+ cells from lymphoid tissue rather than regeneration of CD4+ cells in the setting of viral suppression. A multidose study demonstrated continued safety over an extended treatment period and provided data on the development of ibalizumab resistance. Resistance testing showed reduced susceptibility relative to baseline. Resistant isolates remained dependent on CD4 for viral entry, suggesting that resistance did not develop through the use of alternative receptors. Genotypic analysis was unable to identify mutations, diagnostic of ibalizumab resistance. Consistent with the allosteric mechanism of ibalizumab's anti-HIV-1 effect, the development of resistance is associated with a reduction in the maximum percentage inhibition rather than the shift in the IC50 characteristic of competitive inhibitors [89,90]. The half-life of IgGs under normal physiological circumstances is two to three weeks [91]. In contrast, the average half-life of ibalizumab is 3 to 3.5 days [89]. This is consistent with observations of other anti-CD4 antibodies, in which internalization or shedding of the receptor results in more rapid antibody degradation. A randomized, double-blind, placebo controlled, phase IIa study has evaluated the ibalizumab efficacy, the results showing a considerable viral load reduction with respect to the placebo arm [92].

CCR5 is a chemokine receptor that mediates activation and migration of T cells and other leukocytes. CCR5-using (R5) viruses typically mediate transmission and then predominate through the progression to symptomatic disease. Viruses can use an alternative chemokine receptor, CXCR4, either exclusively or in addition to CCR5. The CXCR4-using virus can be present initially, but tends to result in an increasing proportion of subjects in the later stages of the disease [93]. CCR5 co-receptor antagonists represent an emerging antiretroviral treatment class and the first to target a host molecule.

Currently, two anti-CCR5 mAbs are being investigated. One of these is CCR5mAb004, a fully human IgG4 monoclonal antibody with robust activity against a diverse panel of HIV-1 isolates; it synergizes *in vitro *with other ARV classes and appears safe and effective in reducing HIV viral load. High levels of receptor occupancy were observed for 14 to 28 days with the highest dose cohorts, suggesting the potential for weekly, fortnightly or even monthly dosing [94].

The other anti-CCR5 mAb is PRO 140, a humanized mAb that also synergizes with small-molecule CCR5 antagonists in laboratory studies [95]. PRO140 is being investigated in two modes of administration: the classical intravenous (IV) form, and subcutaneous (SC) form. The trial involving SC administration is the first to bear the proof of concept for a mAb administered subcutaneously in HIV-1 infected subjects as a potent and long-acting antiretroviral agent.

An IV form of PRO 140 tested as monotherapy in HIV-1 subjects with only R5 virus detectable [96] demonstrated potent and prolonged antiviral activity, with a 1.83 log10 mean reduction in HIV-1 RNA and safety relative to placebo. The successive randomized, double-blind, placebo-controlled IIa trial examined the antiviral activity, tolerability and pharmacokinetics of single intravenous infusions of up to 10-mg/kg of mAbs. All PRO 140-treated subjects treated with 10 mg/kg experienced a 1-log10-unit reduction in HIV-1 RNA level, there being just one exception; a post-study analysis using the enhanced-sensitivity Trofile assay determined that this subject had dual/mixed virus at screening. There was no change in co-receptor tropism or emergence of PRO 140-resistant virus during the course of this study, supporting the view that PRO 140 broadly inhibits R5 HIV-1 with a high barrier to resistance. The maximum tolerated dose of IV PRO 140 has not been determined, suggesting a sizeable margin of safety for PRO 140 SC administration study [97].

The study involving PRO 140 SC administration showed virologic suppression between successive doses and no changes in R5 viral susceptibility to PRO 140 following three weeks of monotherapy, indicating no adaptation of virus to use CCR5 in the presence of drug. Pharmacokinetic data suggest the possibility of a drug regimen administered fortnightly for HIV infected individuals. Proteins and other macromolecules drain from SC sites into both blood capillaries and the lymphatic system. In animals, proteins with molecular weights of greater than 16,000 daltons have been observed to drain primarily into the lymphatic system following SC administration [98]. Such proteins transit through lymph fluid and typically are not absorbed significantly into the blood until they reach the thoracic duct. Since the molecular weight of PRO 140 is approximately 150,000 daltons, a substantial amount of SC PRO 140 can be expected to drain into the lymphatic system and potentially encounter CCR5+ cells in lymphoid tissues prior to reaching the bloodstream. For these reasons, serum concentrations may not provide a full picture of the overall exposure following SC dosing of PRO 140. SC infusion is currently used by individuals with primary immunodeficiency to self-administer at home significantly larger amounts (approximately 11 grams) and volumes (approximately 70 mL total, up to 15 mL/site) of the weekly SC-administered immunoglobulin [99]. Self-administration of 324 mg SC PRO 140 would be much simpler in comparison. Therefore, SC PRO 140 offers the potential for significant dose-dependent HIV-1 RNA suppression and may offer greater convenience for many patients in terms of patient self-administration [100].

The SC injection mode was chosen in order to evaluate PRO140 safety and efficacy as an adjunct to an oral antiretroviral regimen in HIV-infected injection drug users with viral rebound and documented poor adherence to the previous antiretroviral regimen. Therefore, a phase IIb, national, multicenter, randomized, double-blind, placebo-controlled study was initiated and is currently recruiting participants. Given the complications that arise from the occurrence of drug resistances, the use of antibodies together with combined therapy increases the drug number and, therefore, the therapeutic opportunities. In particular, in the case of CCR5 inhibitors, one report has demonstrated that resistance to CCR5 inhibitors may increase the sensitivity of the resistant virus to certain neutralizing antibodies [101].

Compared to CCR5, CXCR4-based blocking agents as therapy against HIV are less attractive due to the crucial role of CXCR4 in many biological processes, and the absence to date of known naturally occurring mutations leading to the inactivation of CXCR4 gene in humans. Moreover, one major problem is linked to the fact that, whereas R5 viruses are found on their own in 50% or more of patients, viruses that using CXCR4 co-receptor (X4) usually are present mixed together with R5 viruses; therefore, the use of CXCR4 specific mAbs could result in only little or transient effect on the overall viremia, also complicating the evaluation of pharmacological activity. However, antibodies against CXCR4 might still provide some benefits for some HIV positive patients when co-administrated with CCR5 antagonists, if the safety of such combinations is established [93].

There is a pressing need for antiviral agents that are effective against multiple classes of viruses. Broad specificity might be achieved by targeting phospholipids that are widely expressed on infected host cells or on viral envelopes. Phosphatidylserine (PS), the most abundant anionic phospholipid of the plasma membrane, is segregated at the inner leaflet of the plasma membrane of resting mammalian cells. Loss of PS asymmetry occurs during apoptosis, cell injury, cell activation and malignant transformation, and results from inhibition of the translocases or activation of PS exporters, or lipid scrambling enzymes, such as scramblases. After enveloped viruses replicate within the host cell, they create their 'envelope' by carrying along part of the host cell's membrane upon exiting. As a result, the target phospholipid becomes exposed on the surface of the virus as well as on the infected host cell [102].

Bavituximab is the first in a new class of patented antibody therapeutics that target and, preferentially bind, to these exposed phospholipids. It has demonstrated broad therapeutic potential across multiple oncology indications and represents a new approach to treating viral disease, too. Bavituximab is currently being evaluated in randomized phase II clinical trials for non-small cell lung cancer and pancreatic cancer, for therapy of chronic HCV infection and for HIV/HCV co-infection. The therapeutic effect of bavituximab appears to be due to ADCC of tumor and virus-infected cells. Since PS exposure is an early event during virus infection, ADCC may limit virus spread. Furthermore, in the infectious disease setting, bavituximab causes opsonization and clearance of infectious virus from the bloodstream, leaving less virus to infect other tissues. Three completed phase I HCV clinical trials have shown that bavituximab is generally safe and well-tolerated. Reductions in serum HCV RNA levels were also observed. A randomized phase II clinical trial with previously untreated HCV genotype-1 infected patients was designed to determine the early virologic response (EVR) rate after 12 weeks of therapy with bavituximab in combination with the antiviral drug ribavirin and safety profile versus pegylated IFN-α-2a and ribavirin. The results show that the combination of bavituximab with ribavirin has a better safety profile than an IFN-containing regimen. However, the EVR development in the bavituximab-containing arm was later than the IFN-containing group; therefore, a longer-term evaluation is needed to adequately compare their effectiveness. In addition, the lower dose level appears to be more active in HCV patients than the high dose does. Such results suggest that future studies evaluating longer bavituximab treatment durations at or around the lower dose level in combination with ribavirin and potentially direct acting antivirals in certain patient populations may hold promise as IFN-free HCV therapeutic regimens [103].

Targeting PS on cells infected with multiple different viruses and on virions themselves is a promising antiviral strategy. Although resistance has developed in monotherapy trials with ibalizumab (an anti-CD4 antibody), host-derived antigen, such as anionic phospholipids, on virus-infected cells are independent of the viral genome and as a consequence the acquisition of drug resistance should be theoretically less problematic than with agents that target virus-encoded components.

#### Immunomodulatory mAbs

Since the discovery of PD-1 as an inhibitory receptor associated with T-cell dysfunction, the roles of various inhibitory receptors on virus-specific CD8+ T cells have been extensively studied in human chronic viral infections, such as HCV, HBV and HIV infections. As blocking the inhibitory receptors *in vitro *restores the functions of virus-specific T cells, novel HIV and HCV treatments based on blockade of several immune checkpoint molecules are being investigated. In particular, mAbs interfering with two major inhibitory networks of the B7:CD28 family, namely the PD-1 and CTLA-4 pathways [104], are currently being studied in clinical trials, to evaluate their safety and efficacy. These mAbs recognize the PD-1 or CTLA-4 receptor and neutralize the binding with their respective ligands.

The PD-1:PD-L1 pathway delivers inhibitory signals which regulate T cell activation. As a result it performs a key role in various processes, namely in multiple tolerance checkpoints that prevent autoimmunity, in the suppressive tumor microenvironment, in the immune-mediated tissue damage, in host defenses aimed at eradicating microbial pathogens and tumors and finally, in T cell exhaustion that contributes to both lack of viral control during chronic infections and to T cell unresponsiveness [105]. In cancers, a strong correlation between increased PD-L1 expression on tumors and a negative survival prognosis in patients has already been observed. Various studies indicate that mAbs targeting the PD-1 signaling pathway reinvigorate antigen-specific T-cell responses and promote an immune response to fight tumors [106]. In HCV infection the relationship between the PD-1 expression and the outcome of the acute HCV infection was questioned; subsequently, recent studies have shown that the progression of acute HCV infection to the chronic stage is associated with a high level of PD-1 on HCV-specific CD8+ T cells, whereas the clearance of HCV infection is associated with lower levels of PD-1 expression [36].

Given these premises, MDX-1106, a fully human antibody also known as ONO-4538, and CT-011, a humanized antibody, both interacting with PD-1 receptor, are being developed as a treatment for cancer disease and for therapy of chronic HCV infection [107]. To date, most clinical experience with PD-1 blockade has been gained with MDX-1106 in the tumor setting. Drug-related grade 3 or 4 toxic effects occurred in 14% of patients, in whom there were drug-related adverse events of special interest, those with potential immune-related causes; they included pneumonitis, vitiligo, colitis, hepatitis, hypophysitis and thyroiditis. Pneumonitis (3%) ranged from isolated radiographic abnormalities to progressive, diffuse infiltrates associated with clinical symptoms in a small number of patients. Although three deaths occurred, mild-to-moderate pneumonitis was managed successfully with either observation or glucocorticoids. However, objective responses were observed in approximately one in four to one in five patients with non-small-cell lung cancer, melanoma, or renal-cell cancer; overall, an adverse-event profile does not appear to preclude its use [108]. Besides these studies, an ongoing phase I safety trial with active hepatitis C genotype 1 infected patients has been designed to assess the safety and tolerability profile of MDX-1106 [109]. Clinical studies to evaluate the use of CT-011 in HCV disease have also been initiated [110].

CTLA-4 is up-regulated on activated T cells and inhibits T cell activation by reducing the production of IL-2 and arresting cell cycle progression. CTLA-4 has also been shown to have an impact on T cell responses in animal tumor models and humans [111,112]. Human trials that used a blocking anti-CTLA-4 mAb demonstrated a reduction in tumor mass and clinical benefit in a substantial minority of treated subjects. Studies of the role for CTLA-4 in chronic infections have produced mixed results. In chronic HIV infection, many studies indicate that impaired CD4+ T cell function is associated with viral persistence [113], although the function of CTLA-4 in causing HIV persistence by suppressing T cell function remains unclear [114]. On the other hand, CTLA-4's role in chronic HCV infection seems to be more defined. The HCV-specific CD8+ T cells found in the livers of chronic HCV patients overexpressed not only PD-1, but also CTLA-4. Co-expression of PD-1 and CTLA-4 was observed in liver-infiltrating lymphocytes, but not in peripheral blood lymphocytes [36], suggesting the phenotypic differences of virus-specific CD8+ T cells in different *in vivo *compartments. PD-1 and CTLA-4 expressing HCV-specific T cells were profoundly dysfunctional [115].

Tremelimumab is a fully human IgG2 mAb directed against CTLA-4. While a phase II study for HIV disease with this drug has been withdrawn prior to enrollment, clinical trials for HCV disease are still underway. Tremelimumab binds to activated T lymphocytes and results in inhibition of B7-CTLA-4-mediated down-regulation of T-cell activation. It also acts as an IL-2 stimulant. It was generated, using XenoMouse technology (Figure 2), as an anticancer agent and is currently in worldwide phase III development for malignant melanoma, phase II development for colorectal cancer, gastrointestinal cancer, gynecological cancer and non-small cell lung cancer in the US and other countries. It is also being investigated for prostate, breast and pancreatic cancer in various countries. As for anti-PD-1 antibodies, immune-related adverse effects of tremelimumab are of special interest because of its presumed mechanism of action. Most of the experience in identifying and managing CTLA-4 treatment-related side effects has derived from studies in cancer, particularly in melanoma. These effects mainly include colitis/diarrhea, dermatitis, hepatitis and endocrinopathies; uveitis, nephritis and inflammatory myopathy also have been occasionally reported. These unique side effects are likely a direct result of breaking immune tolerance upon CTLA-4 blockade; they are generally mild, reversible and manageable, following specific treatment guidelines that include symptomatic therapies or systemic corticosteroids [116]. In December 2008, Pfizer initiated a phase II trial in patients with late-stage unresectable liver cancer who also have hepatitis C infections. The primary endpoint of this single-armed study is the ability of tremelimumab to produce tumor responses among HCV-infected patients with hepatocellular carcinoma and to produce changes in hepatitis C viral load. The first results indicate that tremelimumab demonstrated an excellent safety profile, with a promising antitumor efficacy against HCC in 17 patients, as well as an intense antiviral activity. In fact, a significant and progressive decline in serum HCV viral load was observed, this being associated with an increase in anti-HCV immune response in 76% of patients [117].

Since there are multiple levels of immunoregulation, a synergistic use of antibodies against different checkpoint molecules might represent the next stage in immunotherapy for chronic infectious diseases, as evidenced from *ex vivo *studies about the combined PD-1/CTLA-4 blockade in HCV disease [36]. Furthermore, because the host mechanisms that inhibit T cell activity are common and conserved aside from specific virus-encoded immune evasion strategies, the antibodies targeting inhibitory receptors may prove extremely versatile drugs potentially effective against multiple classes of viruses.

## Conclusions

The need to treat HIV and HCV infectious diseases, two epidemics of global impact, has reawakened interest in mAb-based therapy, supporting a variety of clinical studies. The results that are emerging, will help to create models for the further development of such drugs and extend their use against other viruses as well.

Although the mAb production costs are high, increasing advances of biotechnology and production systems will make them more competitive on the market, and new approaches, such as using mAb cocktails or combining mAbs with available drugs, will improve effectiveness. Treatment with mAbs as part of a drug regimen is the most likely future for mAbs that block HCV and HIV infection in order to avoid viral escape, while chronic treatment could attract further investments from pharmaceutical companies. Furthermore, broad spectrum mAbs, such as bavituximab and immunomodulatory mAbs, could be useful against a whole range of diseases, thus extending marketability and profit margins.

This review has focused on the use of intact mAbs as a novel emerging and versatile class of pharmaceuticals. It is important to note, however, that biotechnology also provides the opportunity to build various antibody formats whose improved pharmacokinetics and pharmacodynamic properties could be co-opted in the fight against infectious diseases.

## Abbreviations

ADCC: antibody-dependent cell-mediated cytotoxicity; CDC: complement-dependent cytotoxicity; CHO-Cell: Chinese Hamster Ovary cell; CMV: cytomegalovirus; CRS: cytokine-release syndrome; CTLA-4: cytotoxic T lymphocyte antigen 4; Fab: fragment antigen-binding; Fc: fragment crystallizable; FcαRs: receptors for the Fc segment of IgA; FcγRs: receptors for the Fc segment of IgG; GITR: glucocorticoid-induced TNF receptor; HAV: hepatitis A virus; HBV: hepatitis B virus; HCV: hepatitis C virus; HIV: human immunodeficiency virus; IFN: interferon; Ig: immunoglobulin; IL: interleukin; IV: intravenous; LAG-3: lymphocyte activation gene 3; LCMV: lymphocytic choriomeningitis virus; LVP: LipoViral-Particles; mAbs: monoclonal antibodies; MHC: major histocompatibility complex; nAb: neutralizing antibody; NK: natural killer cell; PD-1: programmed cell death 1; PS: phosphatidylserine; R5: viruses that use only the CCR5 co-receptor; RSV: respiratory syncytial virus; SC: subcutaneous; TCR: T cell antigen receptor; T_EM _cells: effector memory T cells; TGFβ: transforming growth factor β; TIM3: mucin domain protein 3; TNF: tumor necrosis factor; Treg cells: immunosuppressive regulatory T cells; VZV: vesicular stomatitis virus; X4: viruses that use only the CXCR4 co-receptor.

## Competing interests

The authors declare that they have no competing interests.

MC is the author of a patent about a mAb against P-glycoprotein (patent number: 6063621), but this mAb is not suitable for clinical applications in HIV and/or HCV diseases

## Authors' contributions

MF and AA contributed equally to writing and editing the manuscript. MC and SV critically reviewed the manuscript and made final changes. All authors have read and approved the final version of the manuscript.

## Authors' information

MF, after her Doctorate (Immunology, 2006, University of Rome, Tor Vergata, Italy), was enrolled as a researcher at the Department of Therapeutic Research and Medicine Evaluation at the Istituto Superiore di Sanità, Rome, Italy (ISS). She has acquired extensive expertise in biotechnologies, such as the isolation, characterization and genetic manipulation of human mAbs in single chain format for the development of biological constructs designed for clinical use.

AA, since after his Doctorate in Genetics and Molecular Biology (2005, University of Rome, La Sapienza, Italy), was enrolled as a researcher at the Department of Therapeutic Research and Medicine Evaluation at the ISS, where he has developed extensive skills in biotechnological strategies for the genetic manipulation of recombinant antibodies in order to design constructs suitable for clinical applications.

MC, since 2003, has been the Director of the Section of Pharmacogenetics, Drug Resistance and Experimental Therapeutics of the Department of Therapeutic Research and Medicine Evaluation at the ISS; he is an Expert Member of EMEA, Pharmaeuropa and ISS for the evaluation of biotech derived products and for GMP inspection. His research activities are mainly aimed at elucidating the complex phenomenon of multidrug resistance (MDR) through the identification of specific mAbs.

SV is currently the Director of the Department of Therapeutic Research and Medicine Evaluation at the ISS. The main research interests of Dr Vella are as follows: the development of antiretroviral therapy; the study of HIV resistance to antiretroviral drugs; the mechanisms of immune reconstitution; and, finally, operational and implementation research in resource limited settings. He chaired or participated in many international clinical studies on antiretroviral therapy, and he is currently the coordinator of the - European Commission-funded HIV Clinical Trials Network (NEAT).

## Pre-publication history

The pre-publication history for this paper can be accessed here:

http://www.biomedcentral.com/1741-7015/11/4/prepub
